# Discovery of a novel target for cancer: *PRR14*

**DOI:** 10.1038/cddis.2016.401

**Published:** 2016-12-01

**Authors:** Shaowen Xiao, Mei Yang

**Affiliations:** 1 Department of Radiotherapy, Beijing University Cancer Hospital, Beijing, China; 2 Second Xiangya Hospital, Central South University, Changsha, Hunan, China

Gross changes in nuclear morphology are a standard criteria used in the diagnosis of many cancers. Cancerous cells are characterized by enlarged nuclei and prominent nucleoli. However, the specific nuclear components involved, the underlying mechanisms, and the implications for cancer progression, are far from clear. The nuclear lamina, a scaffold-like network of protein filaments surrounding the nuclear periphery, is required in the regulation of most nuclear activities, including nuclear shape, DNA replication, RNA transcription, nuclear and chromatin organization, cell cycle regulation, cell development and differentiation, and apoptosis *et al.*^1^ During tumorigenesis, the components of the nuclear lamina undergo significant alterations and are suggested biomarkers in certain types of cancer, such as lamin A/C in colorectal cancer^2^ and lamin B1 in liver cancer.^3^ It can therefore be hypothesized that the nuclear lamina plays a key role in connecting nuclear morphologic change with its functional alteration during tumorigenesis.

*PRR14* has previously been identified as a novel component of the nuclear lamina, tethering heterochromatin to the nuclear lamina during the cell cycle.^4^ Our group confirmed this observation in myoblast C2C12 cells and demonstrated that this bridge is not only physical but functional as well; PRR14 directly binds to HP1α, a constitutive component of heterochromatin, and myogenesis is tightly regulated by this binding.^5^ Disruption of this interaction almost completely blocked myogenesis and the majority of cells underwent apoptosis. Could *PRR14* be a missing link in the common mechanism mediating nuclear morphological change and its functional alteration during tumorigenesis?

In our recent paper published in *Oncogene*, we have shown that the expression level of *PRR14* is significantly increased in lung cancer with, or without, gene copy number variation.^6^ Indeed, *PRR14* is commonly elevated in many cancer types besides lung cancer^7^ (Figure 1a). Elevated expression of PRR14 at the protein level has yet to be confirmed due to a lack of an appropriate antibody. However, in the future, it would be meaningful to monitor PRR14 protein expression levels during the process of tumorigenesis.

From comparative analysis of differentially expressed transcripts, between high-*PRR14* and low-*PRR14* expressing samples, within different types of lung cancer collections in TCGA (lung adenocarcinoma and lung squamous cell carcinoma), we conclude that *PRR14* expression is positively associated with both gene expression and generic transcription pathways. In addition, PRR14 overexpression results in markedly enlarged cell size. Thus, we hypothesized that *PRR14* may be a novel oncogene activating the PI3-kinase/Akt/mTOR signal pathway, which is known to regulate mammalian cell size through generic gene expression^8, 9^ and a commonly activated pathway in lung cancer.^10^ Consistent with this, the high expression of *PRR14* significantly associates with a decreased 5-year survival rate in more than 1900 lung cancer patients. And the PI3K/Akt/mTOR pathway activation was confirmed through analysis of phosphorylation status of its downstream effectors. Furthermore, we demonstrated that the activation of the pathway was achieved by direct interaction of PRR14 with GRB2, a regulator of PI3K signaling pathway.^11, 12^ Consequently, elevated PRR14 promoted, and reduced PRR14 impeded, lung cancer cell proliferation as well as tumor formation. As expected, elevated PRR14 was specifically associated with a heightened sensitivity to the inhibitors of PI3K (GDC-0941) and its downstream effector mTOR (Torin2 and LY294002), but showed no effect on the mitogen-activated protein kinase (MAPK) inhibitor (U0126; Figure 1b). Fortunately, we got two *PRR14* variants with higher affinity for GRB2 in tumors and they demonstrated a higher capacity to activate PI3K/AKT/mTOR signaling pathway and form tumors, which further confirmed our hypothesis.

GRB2 is also involved in Ras signaling pathway, and AKT/mTOR and MAPK pathways are both its downstream signal pathways.^13^ To exclude the possibility that PRR14 activates AKT/mTOR pathway through Ras instead of PI3K pathway, the phosphorylation status of ERK was used as a readout of the MAPK pathway activity, an alternate downstream pathway of Ras from the Akt/mTOR pathway. The phosphorylation status of ERK was not significantly altered.

The PI3K pathway has been demonstrated to regulate diverse cellular processes including proliferation, adhesion, survival and motility, and its aberrant activation is frequently observed in various cancers conferring sensitivity and resistance to conventional therapies. Therefore, the PI3K pathway is considered a well-validated target for cancer treatment.^14^ Although the activity of the pathway has been extensively explored, previous research has mainly focused on the cytoplasmic component, whereas its functions and effector proteins within the nucleus are poorly understood. Noticeably, both previous^4^ and our^5^ research has demonstrated that PRR14, with nuclear localization signal in both its N- and C-terminals, is predominately localized within the cell nucleus including the nuclear lamina during mitosis. Our findings may therefore provide a novel and common nuclear target in the PI3K pathway.

## Figures and Tables

**Figure 1:**
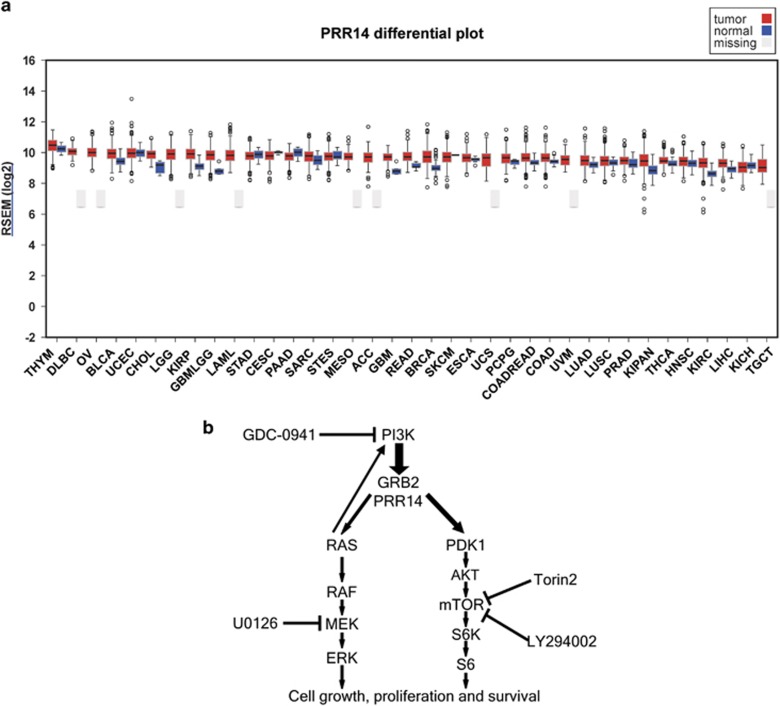
(**a**) *PRR14* mRNA expression level in different types of cancer and their healthy counterparts was extracted from TCGA data and analyzed by firebrowse (http://firebrowse.org/): ACC, adrenocortical carcinoma; BLCA, bladder urothelial carcinoma; BRCA, breast invasive carcinoma; CESC, cervical squamous cell carcinoma and endocervical adenocarcinoma; CHOL, cholangiocarcinoma; COAD, colon adenocarcinoma; COADREAD, colorectal adenocarcinoma; DLBC, lymoid neoplasm diffuse large B-cell lymphoma; ESCA, esophageal carcinoma; GBM, glioblastoma multiforme; GBMLGG, glioma; HNSC, head and neck squamous cell carcinoma; KICH, kidney chromophobe; KIPAN, pan-kidney cohort (KICH+KIRC+KIRP)); KIRC, kidney renal clear cell carcinoma; KIRP, kidney renal papillary cell carcinoma; LAML, acute myeloid leukemia; LGG, brain lower grade glioma; LIHC, liver hepatocellular carcinoma; LUAD, lung adenocarcinoma; LUSC, lung squamous cell carcinoma; MESO, mesothelioma; OV, ovarian serous cystadenocarcinoma; PAAD, pancreatic adenocarcinoma; PCPG, pheochromocytoma and paraganglioma; PRAD, prostate adenocarcinoma; READ, rectum adenocarcinoma; SARC, sarcoma; SKCM, skin cutaneous melanoma; STAD, stomach adenocarcinoma; STES, stomach and esophageal carcinoma; TGCT, testicular germ cell tumors; THCA, thyroid carcinoma; THYM, thymoma; UCEC, uterine corpus endometrial carcinoma; UCS, uterine carcinosarcoma; UVM, uveal melanoma. (**b**) A schematic representation of *PRR14*'s role in PI3K/Akt/mTOR signaling pathway
